# Pre-Transplant Immune Dysregulation Predicts for Poor Outcome Following Allogeneic Haematopoietic Stem Cell Transplantation in Adolescents and Adults with Inborn Errors of Immunity (IEI)

**DOI:** 10.1007/s10875-024-01854-y

**Published:** 2025-01-06

**Authors:** Thomas A. Fox, Valerie Massey, Charley Lever, Rachel Pearce, Arian Laurence, Sarah Grace, Filippo Oliviero, Sarita Workman, Andrew Symes, David M. Lowe, Valeria Fiaccadori, Rachael Hough, Susan Tadros, Siobhan O. Burns, Markus G. Seidel, Ben Carpenter, Emma C. Morris

**Affiliations:** 1 UCL Institute of Immunity and Transplantation, UCL, London, UK; 2 Department of Haematology, University College London Hospitals NHS Foundation Trust, London, UK; 3 Department of Immunology, Royal Free London Hospitals NHS Foundation Trust, London, UK; 4 Kings College London, London, UK; 5 Division of Pediatric Hematology-Oncology, Department of Pediatrics and Adolescent Medicine, Styrian Children’s Cancer Research Unit for Cancer and Inborn Errors of the Blood and Immunity in Children, Medical University of Graz, Graz, Austria

**Keywords:** Inborn errors of immunity, Stem cell transplantation

## Abstract

Allogeneic haematopoietic stem cell transplantation (alloHSCT) is safe and effective for adolescents and adults with inborn errors of immunity (IEI) with severe manifestations of their disease. The haematopoietic cell transplantation comorbidity index (HCT-CI) score predicts transplant survival in non-malignant diseases, including IEIs. We hypothesised that immune dysregulation pre-transplant may also influence transplant outcomes. We calculated the pre-transplant immune dysregulation and disease activity score (IDDA v2.1) for 82 adolescent and adult IEI patients (aged ≥ 13 years). Three-year overall survival (OS) for the whole cohort was 90% (*n* = 82) with a median follow up of 44.7 months (range 8.4 to 225.8). Events were defined as acute graft-versus-host disease (GvHD) grades II or above, chronic GvHD of any grade, graft failure, or death from any cause. Three-year event free survival (EFS) for the whole cohort was 72%. In multivariable analysis the IDDA v2.1 score pre-transplant and HCT-CI score significantly impacted OS (hazard ratio 1.08, *p* = 0.028) and EFS (hazard ratio 1.04, *p* = 0.0005). Importantly, 35% of this cohort had a high IDDA v2.1 score (≥ 15) and low HCT-CI score (< 3) suggesting that the risks of alloHSCT may be underestimated in a proportion of patients with IEI if the HCT-CI score is used alone. These findings support the potential for improved outcomes following successful modulation of immune dysregulation pre-transplant. The IDDA v2.1 score has utility as an objective measurement of pre-transplant immune dysregulation providing additional information regarding the risks and potential complications of alloHSCT in an individual IEI patient.

**Supplementary Information:**

The online version contains supplementary material available at 10.1007/s10875-024-01854-y.

## Introduction

Inborn errors of immunity (IEIs) are rare inherited disorders of the immune system. Nearly 500 distinct IEIs have been described to date [1]. The clinical phenotype of individual disease entities and their prognosis is often uncertain [2–4]. Allogeneic haematopoietic stem cell transplantation (alloHSCT) has been used as curative therapy for selected IEIs since 1968 [5–8]. The rationale for alloHSCT in severe combined immunodeficiency (SCID) is clear, as survival past infancy requires definitive treatment [8, 9]. In some non-SCID IEIs, such as Wiskott-Aldrich syndrome (WAS) and chronic granulomatous disease (CGD), the evidence in favour of alloHSCT is well established [8, 10–16]. In rarer non-SCID IEIs, transplantation is considered in patients with severe disease manifestations, such as life-threatening infections, treatment-refractory autoimmunity or immune dysregulation, and malignancy [17]. Historically, across all IEIs, increasing age at transplant is associated with poorer outcomes [15, 18, 19].

In recent years, more patients with IEIs are being referred for definitive therapy in adolescence or adulthood. Reasons include an initial mild clinical phenotype, delayed diagnosis or lack of genetic diagnosis, late presentation, improved conservative management or prior difficulties identifying suitable donors [3, 20]. Some patients treated with gene therapy or alloHSCT in infancy or early childhood require a second procedure later in life.

There are increasing data describing good outcomes following alloHSCT in older patients with IEIs conditioned with reduced intensity conditioning (RIC) regimens. Single centre studies have demonstrated overall survival rates above 85% with low rates of graft-versus-host disease (GvHD) [3, 21–23]. A large retrospective multicentre study of 329 transplanted adolescents and adults validated the single centre results [24]. Furthermore, a retrospective matched cohort study demonstrated that alloHSCT prevents the progressive morbidity associated with IEIs in older patients [25]. These data have led to more older patients being referred for alloHSCT.

The haematopoietic cell transplantation comorbidity index (HCT-CI) score predicts transplant survival in non-malignant diseases [24, 26]. In a non-transplant setting, the immune deficiency and dysregulation activity (IDDA v2.1) score was devised to standardise evaluation of immune dysregulation in IEIs [27, 28]. The potential for pre-transplant IDDA v2.1 scores to predict outcome following alloHSCT has not yet been assessed. We hypothesised that patients with poorly controlled immune dysregulation pre-transplant may have reduced overall survival and higher risks of developing graft failure or GVHD due to the adverse effects of systemic inflammation on the bone marrow microenvironment and/or donor T cell mediated alloreactivity [29–31]. Here, in addition to reporting updated outcomes including survival and correction of clinical phenotype following alloHSCT in 82 consecutive adolescents and adults with IEIs, we evaluate the predictive value of both HCT-CI and IDDA v2.1 scores on survival in the whole cohort and for different IEI subtypes.

## Methods

### Study Design and Statistical Analysis

Consecutive patients transplanted at the UCL Centre for Immunodeficiency (encompassing the Royal Free NHS Foundation Trust and University College London Hospitals NHS Foundation Trust) were included. Patients provided written consent as per institutional guidelines. Data from medical notes and the alloHSCT database were retrospectively collected between January and November 2023. Data were censored on 3rd November 2023. Data were analysed using Stata version 18.1 (College Station, VA, USA), Probabilities of overall survival (OS) and event-free survival (EFS) were calculated using the Kaplan-Meier method with groups compared using Cox regression where a p value < 0.05 was considered significant. Multivariable Cox regression was performed using a backward stepwise method, with P > = 0.2 for removal. A cutpoint for IDDA v2.1 was chosen by analysis of Harrell’s C-statistic to determine the point which maximizes the C-index and thus the predictive power of the model. Paired data (e.g. IDDA v2.1 scores pre- and post-transplant) were compared using a Wilcoxon matched pairs signed rank test (assuming non-gaussian distribution). An event was defined as acute GVHD grades II or above, chronic GVHD of any grade, graft failure, or death from any cause. The HCT-CI score was calculated using the published scoring system [26, 32]. The IDDA v2.1 score was calculated according to published methods using a Microsoft Excel spreadsheet pre-populated with relevant formulae [27, 28]. Individual components of the IDDA v2.1 score were calculated using the published severity scale ranked from 0 to 4 (supplementary Table 1).

### Patient Characteristics

82 patients transplanted between 2004 and 2023 were included (8 patients pre-2010, 74 patients post-2010) (see Table 1). Median follow up post-transplant was 44.7 months (range 8.4-225.8). The median age at transplant was 21 years (range 13to 60 years). Patients were grouped by age into two categories, adolescents (aged 13–18 at alloHSCT), *n* = 33, median age 16 and adults (aged 19 years and older at alloHSCT), *n* = 49, median age 28 years. 61 patients were male and 21 female. HCT-CI scores were calculated for all 82 patients whilst IDDA v2.1 scores were calculated for 75 patients (insufficient information available for 7 patients) (Supplementary Table 2). Patients had 30 different IEIs, the most common being CGD with 25 patients in total (18 X-linked, 7 autosomal recessive). Patient and disease demographics are detailed in Table 1.

Two patients required alloHSCT after receiving unconditioned autologous gene therapy (gamma retroviral) for X-SCID in infancy. One patient had incomplete immune reconstitution with immune dysregulation (B cell deficiency requiring immunoglobulin replacement, T cell lymphopenia, autoimmune hepatitis, skin rashes with lymphocytic infiltration and frequent respiratory tract infections). The other patient developed T cell acute lymphoblastic leukaemia 2 years post-gene therapy due to insertional mutagenesis. This leukaemia was successfully treated with chemotherapy. However aged 12 he developed immune dysregulation with cytopenias, hepatosplenomegaly, transaminitis and respiratory tract infections and was referred for alloHSCT.

**Table 1:** Patient and disease demographics

Characteristics (Total cohort *n* = 82)	ADOLESCENT (13–18 yrs) *n* = 33 (%)	Adults (≥19 yrs) *n* = 49 (%)
Age at transplant, median (years)	16 (range 13–18)	28 (range 19–60)
Sex		
Male	26 (78)	35 (71)
Female	7 (21)	14 (29)
Median follow-up after transplant (months)	45.2 (range 5 to 157)	43.0 (range 0 to 225)
IEI subgroup and specific disease		
SCID and CID * Undefined SCID/CID* * CD40L Deficiency* * DOCK8 Deficiency* * IL12 receptor-β deficiency* * IL-2 receptor subunit γ* * Hypoomorphic RAG2*	8 (24) *2* *2* *3* *1* *0* *0*	11 (22) *4* *2* *1* *0* *2* *2*
CID with associated or syndromic features * STAT3-LOF* * WAS*	1 (3) *1* *0*	3 (6) *1* *2*
Predominantly antibody deficiencies * APDS2* * Complex CVID (Genetically undefinied)* *XLA*	2 (6) *2* *0* *0*	4 (8) *1* *2* *1*
Immune Dysregulation defects * ALPS* * HLH (Genetically undefined)* * HLH (compound het prf mutations)* * HLH (prf - /-)* * XLP 1 & 2* * CTLA4 Insufficiency* * CD27 Deficiency*	5 (15) *1* *0* *2* *0* *1* *0* *1*	8 (16) *3* *1* *0* *1* *2* *1* *0*
Phagocyte defects * CGD* * X-CGD* * AR-CGD* * GATA2 Deficiency* * LAD type 1 Deficiency* * Clericuzio Syndrome* * Genetically undefined NK cell Deficiency*	17 (52) *15* *11* *4* *1* *1* *0* *0*	19 (39) *10* *7* *3* *7* *0* *1* *1*
Innate and intrinsic immunity defects * IL12RB1 Deficiency* * STAT1 GOF Immunodeficiency* * CARD9 Deficiency*	0 (0) *0* *0* *0*	3 (6) *1* *1* *1*
Autoinflammatory defects * Deficiency of ADA2*	0 (0) *0*	1 (2) *1*

Keywords: ALPS - autoimmune lymphoproliferative syndrome, APDS2 - activated phosphoinositide 3-kinase δ (PI3Kδ) syndrome, AR-CGD - autosomal recessive chronic granulomatous disease, CARD9 - caspase recruitment domain containing protein 9, CD40L - CD40 ligand, CDG - chronic granulomatous disease, CID – combined immune deficiency, CTLA4 - cytotoxic T-lymphocyte-associated protein-4, CVID - common variable immunodeficiency, DADA2 - Deficiency of adenosine deaminase 2, DOCK8 - dedicator of cytokinesis 8, GATA2 - GATA-binding protein 2, HLH - Hemophagocytic lymphohistiocytosis, IL12RB1 - Interleukin-12 receptor beta 1, IL2RG - interleukin-2 receptor gamma-chain, LAD type 1 - leukocyte adhesion deficiency type 1, NK - natural killer, RAG - recombination-activating gene, SCID – severe combined immune deficiency, STAT1-GOF - signal transducer and activator of transcription 1 gain-of-function, STAT3-LOF - signal transducer and activator of transcription 3 loss-of-function, WAS - Wiskott-Aldrich syndrome, X-CGD - X-linked chronic granulomatous disease, XLA - X-linked agammaglobulinemia, XLP 1 & 2 - X-linked lymphoproliferative disease type 1 and 2

### Transplant Characteristics

#### Stem cell Source

20 patients had 10/10 HLA-matched related donors (18 siblings, 2 fully matched paternal donors), 49 had 10/10 HLA-matched unrelated donors, 13 had mis-matched (all 9/10) unrelated donors. Mobilised peripheral blood stem cells were used in 74 patients whilst bone marrow was used in the remaining 8 patients.

#### Conditioning Regimens

T cell depleted (in vivo alemtuzumab or rabbit anti-thymocyte globulin, ATG thymoglobulin) reduced intensity conditioning (RIC) regimens were used in 81 patients. One patient received a full intensity regimen (for Gata2 related myelodysplasia). For the RIC regimens, doses were as follows: fludarabine (30 mg/m2) with melphalan (140 mg/m^2^), treosulfan (30–42 g/m^2^), or busulfan (1.6 mg/Kg or area under curve [AUC] 60–70 mg⋅h/L). Selected patients with no preexisting organ damage for whom full donor chimerism was desired for disease correction or who were thought to have a higher risk of graft failure underwent conditioning with myeloablative treosulfan (30–42 g/m^2^) and thiotepa (8–10 mg/kg). One patient who had previously received gene therapy for X-SCID received a T replete graft. Details of conditioning regimens and transplant characteristics are shown in Table 2. All patients received ciclosporin +/- mycophenolate mofetil as GVHD prophylaxis. Post-transplant cyclophosphamide was used in two patients (2 9/10 MMUD donors) as additional GVHD prophylaxis.

No significant differences were observed between the adolescent and adult groups with respect to underlying diagnosis, HCT-CI scores, IDDA scores, donor, stem cell source and conditioning regimens.

**Table 2:** Transplant characteristics by recipient age at alloHSCT

Transplant characteristics	Adolescent (13–18 years), *n* = 33 (%)	Adults (≥ 19 years), *n* = 49 (%)
Diagnosis		
* SCID/CID*	*8 (24)*	*11 (22)*
* CID with associated or syndrome features*	*1 (3)*	*3 (6)*
* Antibody defect*	*2 (6)*	*4 (8)*
* Immune Dysregulation defect*	*5 (15)*	*8 (16)*
* Phagocyte defect*	*17 (52)*	*19 (39)*
* Innate and intrinsic immunity defect*	*0 (0)*	*3 (6)*
* Autoinflammatory defect*	*0 (0)*	*1 (2)*
Genetic diagnosis confirmed	30 (91)	40 (82)
HCT CI score pre-alloHSCT, median	1 (range 0–6)	1 (range 0–6)
0–1	18 (54)	28 (57)
2	8 (24)	6 (12)
> 3	7 (21)	15 (31)
IDDA v2.1score pre-alloHSCT, median	15 (range 6–47)	17 (range 5–50)
< 10	7 (21)	5 (10)
10–19	12 (36)	22 (45)
20–29	9 (27)	10 (20)
> 30	4 (12)	6 (12)
NA	1 (3)	6 (12)
Donor		
MRD	8 (24)	12 (24)
MUD	22 (67)	27 (55)
MMUD	3 (9)	10 (20)
Conditioning Regimen		
FMC	4 (12)	34 (69)
FBC	8 (24)	7 (14)
FB-ATG	3 (9)	2 (4)
FTC	11 (33)	2 (4)
Yt-FTT	1 (3)	0
FTTC	5 (15)	3 (6)
FT-ATG	1 (3)	0
FluCy 12GyTBI	0	1 (2)

Keywords: CGD - chronic granulomatous disease, CID - combined immune deficiency, FB-ATG - fludarabine busulfan and anti-thymocyte globulin, FBC - fludarabine busulfan campath, FluCyTBI - fludarabine cyclophosphamide with total body irradiation, FMC - fludarabine melphalan campath, FT-ATG - fludarabine treosulfan with anti-thymocyte globulin, FTC - fludarabine treosulfan campath, FTT - fludarabine treosulfan thiotepa, HCT CI - haematopoietic cell transplantation comorbidity index, IDDA v2.1- immune dysregulation and disease activity, MMUD - mismatched unrelated donor, MRD - matched related donor, MUD matched unrelated donor, SCID – severe combined immune deficiency, Yt-FTT - yttrium coupled CD66 antibody radioimmunotherapy with fludarabine treosulfan and thiotepa

#### Supportive care

Routine antimicrobial prophylaxis was given to all patients according to institutional guidelines. All patients received varicella zoster reactivation prophylaxis with aciclovir. Patients seropositive for cytomegalovirus (CMV) transplanted after July 2019 received letermovir prophylaxis until 100 days post-transplant. Surveillance for CMV, adenovirus and Epstein-Barr virus was performed by polymerase chain reaction, weekly until at least 100 days post-transplant or whilst on immunosuppressive agents. Pre-emptive treatment was administered according to institutional guidelines. For patients receiving immunoglobulin replacement therapy pre-transplant this was continued for a minimum of 6 months post alloHSCT before weaning.

#### Peripheral Blood Chimerism Analysis

Lineage-specific chimerism analysis was performed on peripheral blood mononuclear cells (PBMCs). Chimerism results for whole blood, B cell (CD19), T cell (CD3) and granulocyte (CD15) compartments were reported. Patients with a sex-mismatched donor had chimerism analysis performed by fluorescence in situ hybridization. In sex-matched donors, polymerase chain reaction of short tandem repeats was performed [33].

### Assessment of Vaccine Responses

Normal vaccine responses were defined as the demonstration of protective IgG titres after vaccination. Impaired polysaccharide vaccine responses were defined as patients with normal vaccine response to protein antigen but impaired vaccine response to polysaccharide vaccination (defined as protective antibody titres > 0.35ug/ml to 7/13 serotypes tested in less than 50% of the serotypes tested post vaccination with Pneumovax). Patients labelled as having impaired vaccine response failed to demonstrate protective titres after protein antigen vaccination (either tetanus or H. influenzae B).

## Results

### Overall Survival (OS) and Event-Free Survival (EFS)

**Fig. 1:**
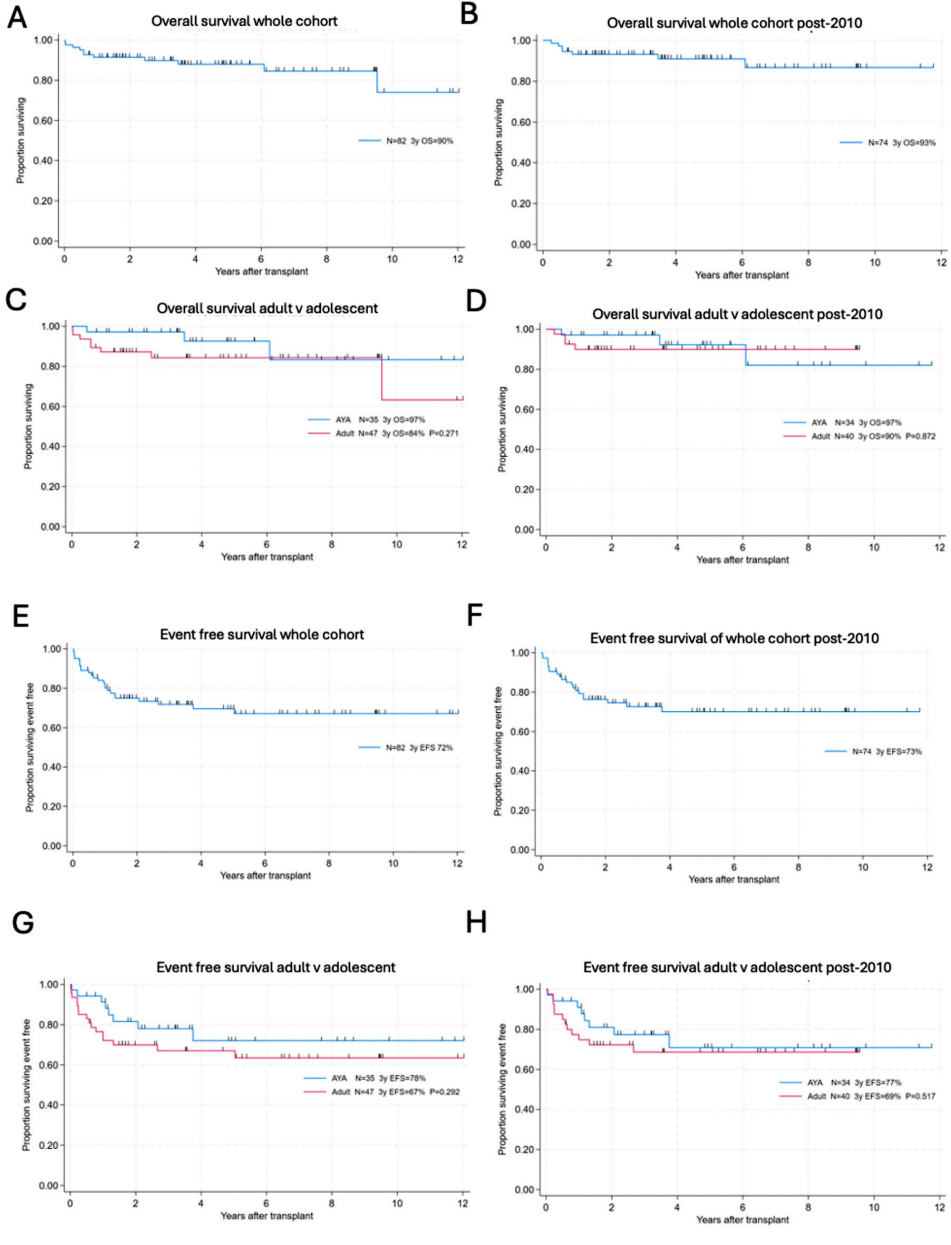
Probabilities of OS and EFS: (**A**) OS whole cohort (*n* = 82), three-year OS = 90%. (**B**) OS whole cohort post 2010 (*n* = 74), three-year OS = 93%. (**C**) OS in the adolescent cohort (3-year OS 97% *n* = 35) and the adult cohort (3-year OS 89% *n* = 47). (**D**) OS in the adolescent cohort post-2010 (3-year OS 97% *n* = 34) and the adult cohort (3-year OS 90% *n* = 40). (**E**) EFS whole cohort (*n* = 82), three-year EFS = 72%. (**F**) EFS whole cohort post-2010, three-year EFS = 73%. (**G**) EFS in the adolescent cohort (*n* = 35), three-year EFS = 78% and the adult cohort (*n* = 47), three-year EFS = 67%. (**H**) EFS in the adolescent cohort post 2010 (*n* = 34), three-year EFS = 78% and the adult cohort (*n* = 40), three-year EFS = 67%

Three-year OS for the whole cohort (*n* = 82) was 90% with a median follow up of 44.7 months (range 8.4 to 225.8) (Fig. 1A). Three-year OS in patients transplanted post-2010 (reflecting our contemporary practice and establishment of a joint adult immunology/HSCT clinic) was 93% (*n* = 74, median follow up 43.6 months, range 8.4 to 138.8 months) (Fig. 1B). No statistically significant difference in OS between the adolescent cohort (3-year OS 97% *n* = 35) and the adult cohort (3-year OS 84% *n* = 47, *p* = 0.271) was observed (Fig. 1 C, 1D). Three-year EFS for the whole cohort (*n* = 82) was 72% (post-2010 *n* = 74 EFS was 73%). Similarly, there was no significant difference between the adolescent and the adult cohort (3-year EFs 78% and 67% respectively, *p* = 0.292) (Fig. 1G, 1 H).

### Pre-Transplant Bridging Therapy

Two patients with targetable genetic mutations continued therapy during the peri-transplant period. The patient with the STAT1 gain-of-function (STAT1 GOF) immunodeficiency continued tofacitinib, whilst the patient with CTLA-4 insufficiency continued abatacept. Patients with HLH continued their immunomodulatory therapy until the start of conditioning. One patient was managed with dexamethasone, anakinra and ciclosporin, one with anakinra alone and one with alemtuzumab. Where possible, corticosteroids were weaned prior to transplant, however 6 patients were still on corticosteroids at the start of transplant conditioning. One X-CGD patient with severe colitis continued ustekinumab until transplant.

### Engraftment

Median time to neutrophil engraftment was 12 days (range 10–30 days, interquartile range 11–13) and platelet engraftment 14 days (range 10–150 days, interquartile range 13–16 days).

### Graft Failure and Second Procedures

There were four cases of graft failure with autologous reconstitution (1 X-CGD, 1 CTLA-4 insufficiency, 1 Hyper IgE (STAT3 loss-of-function), 1 APDS2/aPI3K syndrome). Graft failure occurred in the X-CGD patient in the context of poor compliance with immunosuppressive therapy post-transplant. The other three patients developed graft failure despite therapeutic immunosuppression. They all had high IDDA v2.1scores pre-transplant (IDDAv2.1 scores of 20, 22.5, and 50).

A further patient (STAT1 GOF immunodeficiency) had poor graft function in the context of pre-existing massive splenomegaly and received a CD34 + stem cell top up. This patient died 319 days post-transplant from sepsis and multi-organ failure following splenic embolization. Splenic embolization was performed due to splenomegaly and severe cytopenias.

Two of the four patients with graft failure, were successfully salvaged with a second alloHSCT. Regarding the two patients not salvaged with second alloHSCT, one of these patients had recurrent IEI-related clinical features in the context of autologous reconstitution and are being considered for a second procedure. Another patient died after data censure.

Two patients received donor lymphocyte infusions (DLI) for falling donor chimerism (whole blood and CD3 + cell chimerism). One patient received three doses of escalating DLI (1 × 10^6/Kg, 1 × 10^7/Kg, and 3 × 10^7/Kg) when whole blood donor chimerism dropped below 50% at 4 years post alloHSCT and reverted to full donor chimerism following DLI. The other patient received DLI 8 months post-transplant to prevent graft failure and autologous reconstitution (whole blood donor chimerism 26% prior to DLI). This patient received two doses of escalating DLI (1 × 10^6/Kg and 1 × 10^7/Kg) with no impact on chimerism (persistent mixed chimerism: T cells 23%, granulocytes 10%, B cells 12%, whole blood 12%).

Details of early and late second procedures are shown in Table 3.

**Table 3:** Summary of second procedures during study period

Original alloHSCT	Diagnosis	Second procedure	Indication for second procedure	Time between first and second procedure	Time post second procedure and outcome/status
FMC sib alloHSCT (2007)	X-CGD	**Donor lymphocyte infusions (1 × 10^6/Kg**,** 1 × 10^7/Kg**,** and 3 × 10^7/Kg**	Persistent mixed chimerism	48 months	Alive 92 months post-second procedure. Asymptomatic, no infections. 100% donor chimerism in all lineages.
FMC (60) MUD alloHSCT	Hyper IgE syndrome (STAT3 loss-of-function)	**FTT ATG 10/10 MUD alloHSCT**	Graft failure with autologous reconstitution. Developed left hip septic arthritis during first transplant.	5 months	Alive 71 months post-second procedure. Asymptomatic. 100% donor chimerism all lineages. Off Immunoglobulin replacement.
FMC (30) 10/10 MRD (father) alloHSCT	Undefined CID	**Received 2x DLI for (1 × 10^6/Kg**,** 1 × 10^7/Kg)**	Mixed chimerism	8 months	Alive, 62 months post-second procedure. Asymptomatic. Persistent mixed chimerism (T cells 23%, granulocytes 10%, B cells 12%, whole blood 12%).
BuCyATG 9/10 MUD alloHSCT	Gata2/MDS	Yttrium/FTT 9/10 MMUD alloHSCT	Disease relapse (MDS-EB2).	33 months	Died 39 months post-second procedure. Cause of death: AML.
FBC (60) 10/10 MUD alloHSCT	AR CGD	**Flu Bu ATG 10/10 MUD alloHSCT**	Secondary graft failure	17 months	Died 24 months post second procedure. Cause of death: Grade 4 acute GvHD.
FMC (60) 9/10 MMUD alloHSCT	HLH (no genetic cause identified)	**CD34 + stem cell top up (dose 6.2 × 10^6/kg).**	Cytopenias and poor graft function.	4 months	Alive 45 months post-second procedure. Limited chronic GvHD. Cytopenias improved. No further episodes of HLH.
FBC (60) 10/10 MUD alloHSCT	X-CGD	**Flu Cy TBI (2 Gy) Haploidentical alloHSCT**	Graft rejection (poor compliance with immune suppression)	4 months	Alive. 1 severe lower respiratory tract infection post second alloHSCT. 100% donor chimerism. Ongoing extensive chronic GvHD. Severe bronchiectasis, recurrent RTIs; secondary bacterial infection of skin.
FMC (30) 10/10 MRD alloHSCT	STAT1 gain-of-function	**CD34 + stem cell top up (dose 7.4 × 10^6/kg)**	Cytopenias	3 months	Died 319 days post-transplant from sepsis and multi-organ failure following splenic embolization.

Abbreviations; BMT – bone marrow transplant, FMC – fludarabine, melphalan and campath (alemtuzumab), FTT – Fludarabine, treosulfan and thiotepa, alloHSCT – allogeneic haematopoietic stem cell transplantation, Flu Cy TBI – Fludarabine, cyclophosphamide, total body irradiation, MRD – matched related donor, MUD – matched unrelated donor, MMUD -mis-matched unrelated donor, X-CGD - X-linked chronic granulomatous disease, AR-CGD – autosomal recessive chronic granulomatous disease, DLI – donor lymphocyte infusion, GvHD – graft-versus-host disease

### Peripheral Blood Chimerism

**Fig. 2:**
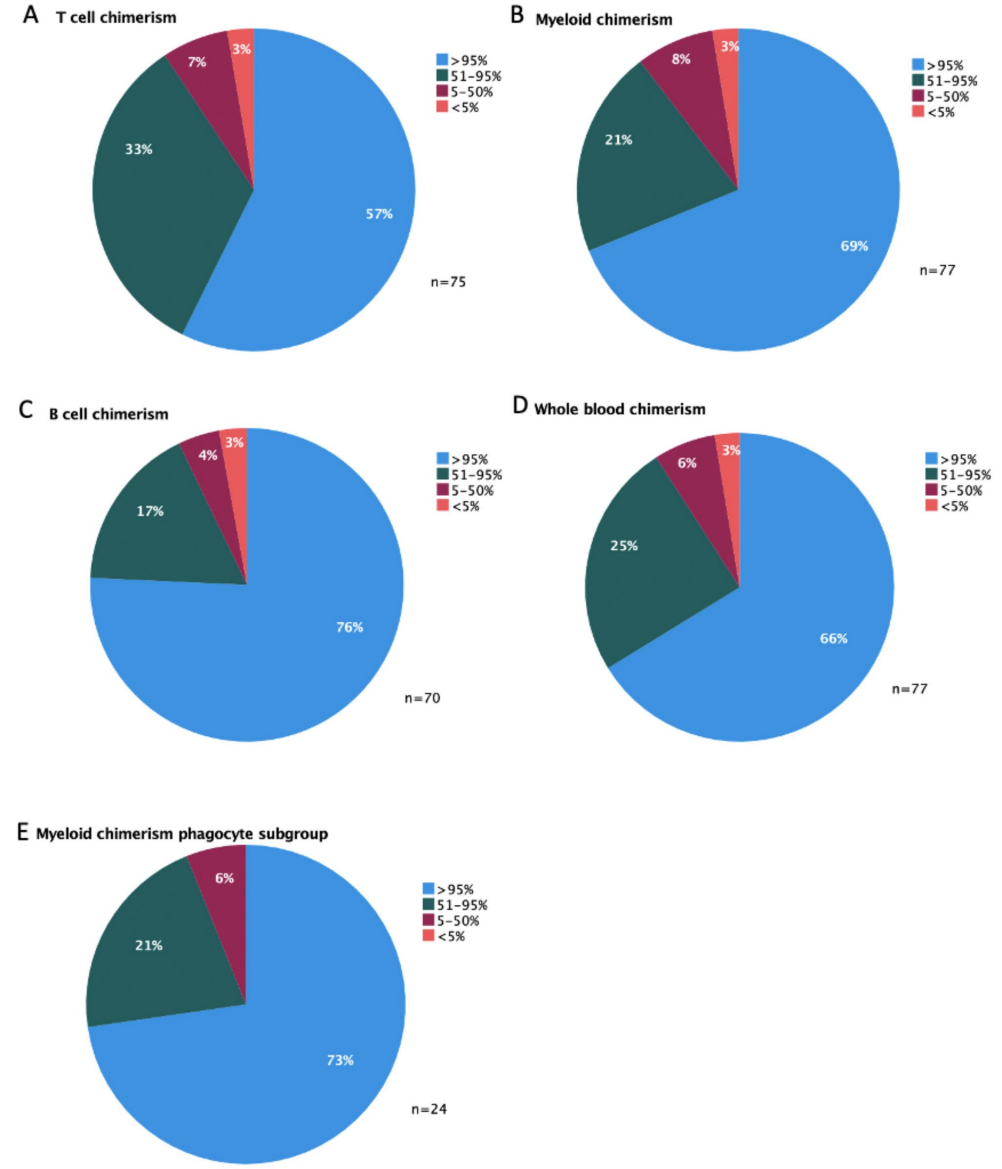
Peripheral Blood Chimerism at last follow-up. A: CD3 + T cell chimerism for all patients; B: CD15 + myeloid cell chimerism for all patients; C: CD19 + B cell chimerism for all patients; D: whole blood chimerism for all patients; E: CD15 + myeloid cell chimerism in the phagocyte deficiency subgroup. Keywords: n- number of patients for which data was available, % - donor chimerism

Chimerism data were available for 77 patients at last follow up (5 patients died before post-transplant chimerism was assessed) (Fig. 2). Chimerism data was not available for patients who died in the peri-transplant period (5 patients). Resolution of disease phenotype occurred despite the presence of mixed chimerism in 43% of patients.

### Immune Reconstitution

Immune reconstitution was assessed at > 12 months post-transplant in surviving patients (*n* = 70). At last follow up 50 (71%) were not receiving immunoglobulin replacement therapy (IgRT) and 20 (29%) were. 2 of the patients on IgRT had graft failure with autologous reconstitution. For those not on IgRT, the median IgG level was 9.9 g/L (normal 7–16 g/L), median IgA 1.7 g/L (normal 0.7–4 g/L) and median IgM 0.8 g/L (normal 0.4–2.3 g/L). Data on lymphocyte subsets were available for all surviving patients. The median CD3 count for the whole cohort on last available results was 0.68 × 10^9^ cells/L and the mean CD4 count was 0.35 × 10^9^ cells/L. Excluding the patients who were < 12 months post-transplant (*n* = 3), 45% of patients had a normal CD3 count (> 0.7 × 10^9^ cells/L) and 60% had a normal CD4 count. Vaccine responses were assessed and results available for 26 patients (excluding patients on IgRT and those less than 3 years post-transplant who had not completed their revaccination schedule), of which 15 had a normal response to vaccination with either polysaccharide or protein conjugate vaccination Table 4.

**Table 4:** Immune reconstitution at last follow-up post-alloHSCT

Immunoglobulins					
	**IgG- Median (range) (*n*** **= 50). Reference range 7.0–16.0 g/L**		**IgA- Median (range) (** ***n*** ** = 50). Reference range 0.7–4.0 g/L**		**IgM- Median (range) (** ***n*** ** = 50). Reference range 0.4–2.3 g/L**
Not on IgRT at last f/u (*n* = 50)	9.9 (3.77–14.96)		1.5 (0-3.6)		0.7 (0.11–2.43)
	IgG (*n* = 20)		IgA (*n* = 20)		IgM (*n* = 20)
On IgRT at last f/u (*n* = 20)	6.4 (3.41–17.7)		0.22 (0-3.2)		0.3 (0-0.67)
**Lymphocyte subsets**					
		**CD3 Median (range)** Normal range 0.7–2.1 × 10^9^/L.		**CD4 Median (range)** Normal range 0.3–1.4 × 10^9^/L.	
SCID + CID (*n* = 20)		0.71 (0.12–2.51)		0.35 (0.065–1.47)	
Antibody defects (*n* = 4)		0.2 (0.101–0.32)		0.06 (0.05–0.08)	
Phagocyte defects (*n* = 33)		0.48 (0.09–1.12)		0.26 (0.05–0.76)	
Autoinflammatory defects (*n* = 13)		2.61		1.05	
**Vaccine response (** ***n*** ** = 27)**					
Normal			16		
Impaired response to polysaccharide vaccines only			9		
Impaired vaccine responses			2		

Immunoglobulins: Median (range) for Immunoglobulin G, A and M levels (g/L). Normal ranges for adults: IgG 7.0–16.0; IgA 0.7–4.0; IgM 0.4–2.3;

Lymphocyte subsets: Median (range) CD3 and CD4 lymphocyte cell counts in patients post-alloHSCT categorised by underlying disease group (× 10^9^/L). Normal ranges for adults: CD3 0.7–2.1; CD4 0.3–1.4 Vaccine responses: Responses to pneumococcal or protein conjugate vaccination post-alloHSCT (normal/abnormal)

### Pre-Transplant HCT-CI and IDDA v2.1Scores Predict Outcome Post alloHSCT

**Fig. 3:**
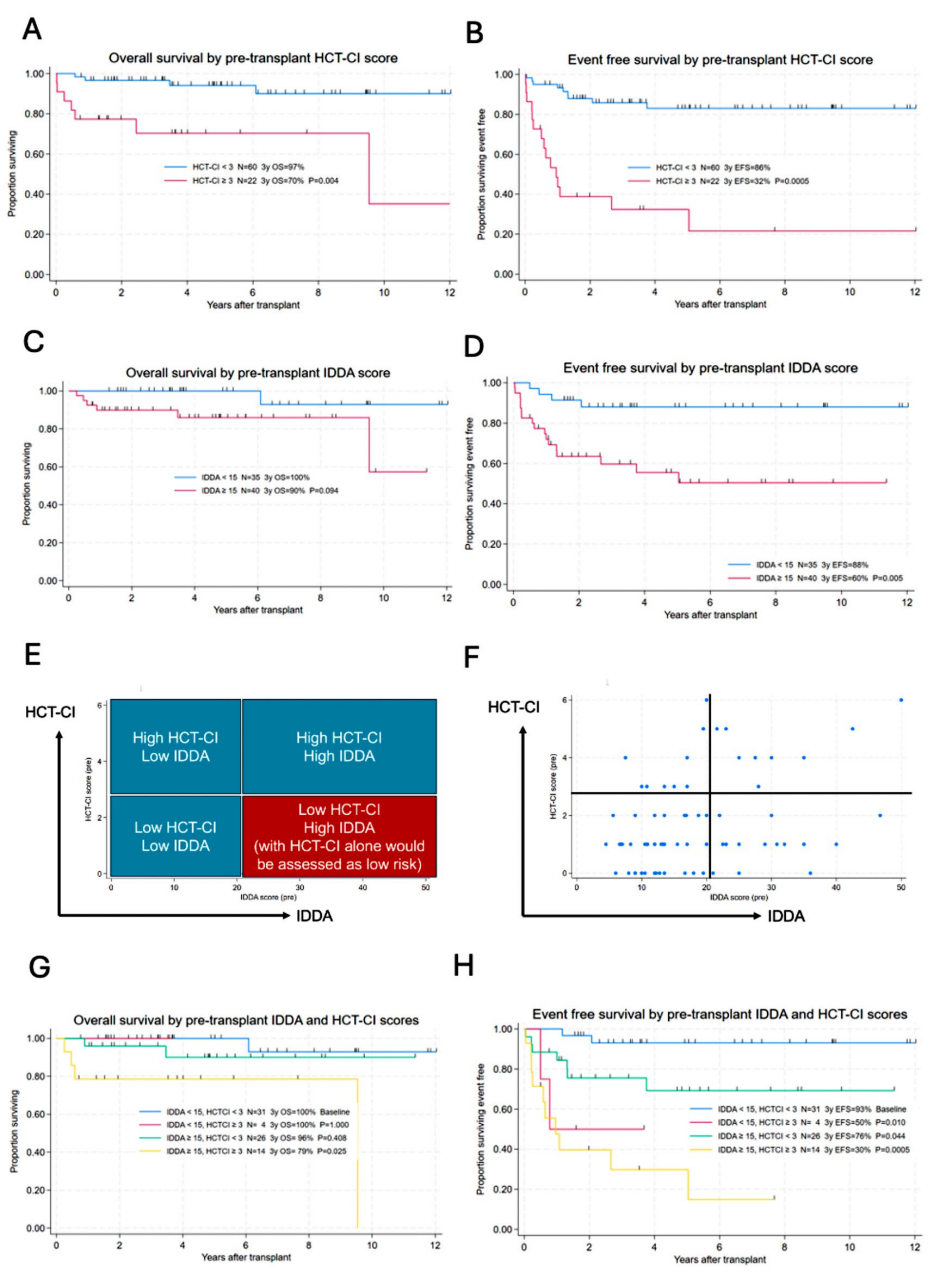
Influence of HCT-CI and IDDA v2.1 scores on OS and EFS: (**A**) 3-year OS; HCT-CI score < 3 = 97%, HCT-CI score ≥ 3 = 70%, *p* = 0.004. (**B**) 3-year EFS, HCT-CI < 3 = 86%, HCT-CI score ≥ 3 = 32% p = < 0.001. (**C**) 3-year OS IDDAv2.1score < 15 = 100%, > 15 = 90%, *p* = 0.094. (**D**) 3-year EFS with IDDA v2.1score < 15 = 88%, > 15 = 60%. (**E**) Schematic demonstrating the cohort of patients for which the IDDAv2.1score would provide additional information to the HCT-CI score in assessing the risk of alloHSCT. (**F**) Distribution of patients (represented by a blue dot) in the cohort by HCT-CI score and IDDA v2.1 score. (**G**) 3-year OS for patients with low IDDAv2.1and HCT-CI scores (*n* = 31), low IDDA v2.1 and high HCT-CI scores (*n* = 4), OS = 100%, *p* = 1.0, high IDDA v2.1 and high HCT-CI scores (*n* = 26) OS 96%, *p* = 0.408 and high IDDAv2.1 and low HCT-CI scores (*n* = 14) OS = 79% *p* = 0.025. (H) 3-year EFS for patients with low IDDAv2.1 and HCT-CI scores (*n* = 31) EFS = 93%, low IDDA v2.1and high HCT-CI scores (*n* = 4) EFS = 50%, *p* = 0.010, high IDDA v2.1 and high HCT-CI scores (*n* = 26) EFS = 76%, *p* = 0.044 and high IDDAv2.1 and low HCT-CI scores (*n* = 14) EFS = 30%, *p* = 0.0005

HCT-CI scores were calculated pre-transplant for all 82 patients. The median HCT-CI score was 1 (range 0–6). 14 patients (17%) had an HCT-CI score of 2 and 22 patients (27%) had an HCT-CI score of 3 or greater. As expected, survival was significantly impacted by HCT-CI score (3-year OS HCT-CI score < 3 97%, HCT-CI score ≥ 3 70%, *p* = 0.004) as shown in Fig. 3A. Event-free survival was similarly impacted by the HCT-CI score (3-year EFS HCT-CI < 3 86%, HCT-CI score ≥ 3 32% p = <0.001) (Fig. 3B). When analysed as a continuous variable there was a statistically significant inverse relationship between HCT-CI score and OS (*p* = 0.016) and EFS (*p* = 0.0005).

The IDDA v2.1 score was calculated for 75 patients pre-transplant. There was insufficient data available to calculate the score for 7 patients (4 CID, 1 CVID, 2 CGD patients, 5 of whom were transplanted pre-2010). The median IDDA v2.1 score pre-transplant was 16.6 (range 6–50). In univariate analysis the IDDA v2.1 score pre-transplant significantly predicted EFS (*p* = 0.005) (Fig. 3D). To identify a useful cutpoint for IDDA v2.1score, Harrell’s C-index was calculated for each possible cutpoint, leading to identification of 15 as the cutpoint of IDDA v2.1 which gave the maximum predictive value.

35% of this cohort (26 patients) had a low HCT-CI score (< 3) but a high IDDA v2.1 score (≥ 15), Fig. 3E, 3 F. These patients would currently be considered low or intermediate risk for transplant (predicted < 21% TRM at 1 year) using the HCT-CI score alone [26]. 3 year EFS for patients with an IDDA v2.1 score < 15 and HCT-CI score < 3 was 93% (95% c.i. 75 − 98%) 3 year EFS for patients with an IDDA v2.1 score ≥ 15 and HCT-CI score < 3 was 76% (95% c.i. 53% 88%) (*p* = 0.043).

In a multivariable analysis of EFS including pre-transplant IDDA v2.1 ≤ 14 / ≥15 and HCT-CI ≤ 2 / ≥3 and the interaction between these prognostic factors, as well as age at transplant and date of transplant, neither IDDA v2.1 (*p* = 0.059, HR = 4.6) nor HCT-CI (*p* = 0.057, HR = 7.1) were independently significant, but the interaction between these two factors combined (HCT-CI ≥ 3 and IDDA v2.1 ≥ 15) was highly significant (*p* = 0.0005, HR = 18.0), Fig. 3H. Likewise in a multivariable analysis of OS including the same variables, the combination of both HCT-CI and IDDA v2.1 (HCT-CI ≥ 3 and IDDA v2.1 ≥ 15) emerges as the only significant factor (*p* = 0.028, HR = 12.6), Fig. 3G.

### Disease Phenotype Reversal Post alloHSCT

**Fig. 4:**
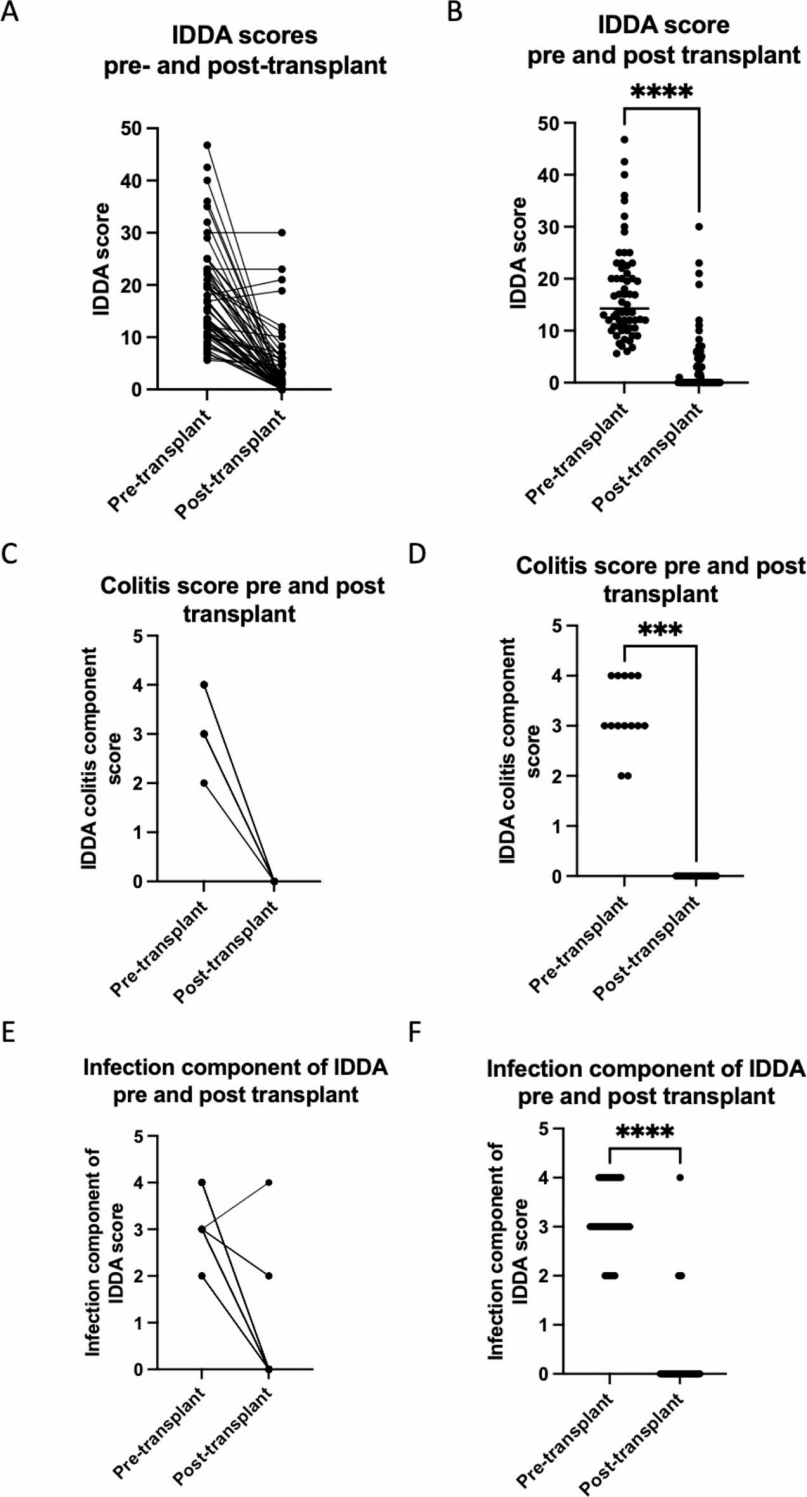
Pre- and post-transplant IDDAv2.1scores: (**A**) Patients with pre- and post- IDDA v2.1 scores (*n* = 60) (pre-transplant: median 14.25, interquartile range 10.73–22.13, post-transplant median 0, interquartile range 0–5). (**B**) Comparison of pre- and post- transplant IDDA v2.1 scores by Wilcoxon matched pairs signed rank test demonstrated a statistically significant difference (*p* < 0.0001). (**C**) Median colitis component of IDDA v2.1 score pre-transplant = 3 (interquartile range 2–4), post-transplant = 0 (interquartile range 0–0). (**D**) Comparison of colitis component of IDDA v2.1 scores (*n* = 14) pre- and post-transplant by Wilcoxon matched pairs signed rank test demonstrated a statistically significant difference *p* = 0.0001. (**E**) Median infection component of IDDA v2.1 score (*n* = 32) pre-transplant median = 3 (interquartile range 3–4), post-transplant median = 0. (**F**) Comparison of infection component of IDDAv2.1scores pre- and post-transplant by Wilcoxon matched pairs signed rank test demonstrated a statistically significant difference *p* < 0.0001

We used the IDDA v2.1 score pre- and post-transplant to assess whether alloHSCT resulted in clinical improvement. The IDDA v2.1 score was calculated at last follow up for all surviving patients (where pre-transplant score was available) and with a minimum of 12 months follow-up (*n* = 60) (post-transplant median IDDA v2.1 14.25, interquartile range 10.73–22.13). We reasoned that the IDDA v2.1 score in the first 12 months post-transplant may be influenced by immunosuppression for GVHD prophylaxis and common early post-transplant complications such as infections, acute GVHD and/or hospitalisations. In all but two patients who had moderate chronic GvHD, the median IDDA v2.1 score was lower post-transplant (median 0, interquartile range 0–5) (*p* < 0.0001) (Fig. 4A, B). Four patients were still receiving systemic immunosuppression when the post-transplant IDDA v2.1 was calculated, and one patient was on anakinra (following HLH recurrence).

Individual components of the IDDA v2.1 score were used to assess the impact of alloHSCT on significant clinical features present prior to transplant (see supplementary Table 1 detailing the components of the IDDA v2.1 score). In all surviving patients with active colitis/inflammatory bowel disease pre-transplant, the colitis component of their IDDA v2.1 score had improved significantly (*n* = 14, *p* < 0.0001) (Fig. 4 C, D) at last follow-up. In surviving patients with severe recurrent infections pre-transplant, a significant difference was observed between pre- and post-transplant scores in the infection component of the IDDA v2.1 score (*n* = 32, *p* < 0.0001) with reduced infections post-transplant. Two patients had persistent infections post-transplant (both had GvHD requiring continued immunosuppression).

## Discussion

Decisions surrounding alloHSCT in older patients with IEI are complex. AlloHSCT outcome data are limited due to the rarity of individual IEIs and by the relatively low number of older patients with IEIs transplanted worldwide. In this study of 82 adolescent and adult patients who have undergone alloHSCT for IEI (29 previously reported with shorter follow-up [3, 24]) we demonstrate excellent 3-year overall survival of 90% adding to the growing body of evidence demonstrating the safety of alloHSCT in older patients with IEI.

We describe, the value of using the IDDA v2.1 score as a measure of pre-transplant immune dysregulation to predict OS and EFS in older IEI patients undergoing alloHSCT. The IDDA v2.1s core quantifies the degree of immune deficiency and immune dysregulation which results from an IEI [27, 28]. Although the IDDA v2.1 score was originally designed to evaluate PIRDs, the updated v2.1 score has been applied to a variety of IEIs to assess the degree of immune dysregulation [27].

We detail resolution of IEI-related clinical features following alloHSCT for 30 different IEIs. Importantly we identify a cohort of patients (a third of patients in this study) who have a low HCT-CI score but high IDDA v2.1 score. This suggests that the risks of graft failure and GVHD following alloHSCT may be underestimated using the existing HCT-CI score alone.

The HCT-CI score has been validated in non-malignant disease settings and outcomes in this cohort are consistent with previously published results that show increasing HCT-CI scores are associated with worse overall survival for alloHSCT for patients with IEIs [24]. The impact of active immune dysregulation as measured by the pre-transplant IDDA v2.1 on alloHSCT outcomes has not been explored and our study suggests that this may be a useful additional tool to help assess an individual’s likelihood of successful outcome following alloHSCT. The IDDA score was significantly associated with adverse outcomes in univariate analysis. In multivariate analysis the IDDA score was not independently significant but statistical significance was observed when combined with HCT-CI score. The lack of independent significance in multivariate analysis is explained by the clear but non-linear correlation between outcome and IDDA score (Fig. 3E, 3 F).

This study has several limitations. It is retrospectively conducted, in a single centre with heterogeneous conditioning regimens in 30 different IEIs. Further work is needed to apply this to a larger cohort that includes paediatric patients to replicate our findings and assess the applicability of our conclusions to a younger cohort.

Our data supports the use of the IDDA v2.1 score to improve individual risk assessment for patients with IEI being considered for alloHSCT. Our study provides further evidence in support of the use of RIC alloHSCT in older patients with IEIs but demonstrates that increased immune dysregulation pre-transplant is associated with increased graft failure and GvHD. The mechanism behind this requires further investigation but increased alloreactivity and/or inflammation in the bone marrow microenvironment may be possible explanations. Interventions aiming to reduce immune dysregulation prior to alloHSCT for IEIs are currently being assessed in a prospective trial (NCT05787574). This study aligns with data from small cohort studies, suggesting that immune dysregulatory diseases such as CTLA-4 insufficiency, LRBA-deficiency, activated PI3K-delta syndrome and STAT1 gain-of-function disorders, may be more difficult to transplant due to higher rates of GvHD and graft failure [28, 34–37]. Future work should focus on how to improve engraftment and reduce rates of GvHD in patients with significant immune dysregulation pre-transplant. Possible strategies include, pre-transplant immune modulatory therapies (e.g. targeted therapies such as abatacept, JAK inhibitors and corticosteroids), optimisation of conditioning regimens (e.g. increasing intensity of conditioning such as FTT regimen for patients with high degree autoinflammation) and autologous gene therapy strategies [38].

Our data have demonstrated that a significant proportion of patients (37%) develop mixed chimerism following RIC alloHSCT for IEIs. Studies suggest this is important for long-term outcomes and is associated with poorer EFS and late complications [39–41]. The degree of chimerm required for phenotype resolution is likely to be disease dependent. Strategies to promote donor engraftment and manage mixed chimerism in the context of alloHSCT for IEIs is an area that requires further study. In contrast to malignant disease where the risk of GvHD following DLI, are more easily justified by the risk of disease relapse, in the IEI setting the occurrence of GvHD is highly undesirable as this results in the patient substituting one immune dysregulatory disease for another. Currently our understanding of the impact of mixed chimerism on long-term outcomes makes decisions regarding DLI (to augment donor chimerism) difficult in the IEI context and our data highlight this as an area for further study.

This is the first study which has explored the impact of the IDDA v2.1 score on transplant outcomes. Whilst these findings need to be reproduced in a larger cohort our data suggest that an increased IDDA v2.1 score has an adverse effect on both OS and EFS. This emphasises the need to attempt to achieve adequate disease control prior to transplant and provides a tool which may be used to aid counselling of IEI patients about their individual risks for alloHSCT.

## Electronic Supplementary Material

Below is the link to the electronic supplementary material.

Supplementary Material 1

## Data Availability

No datasets were generated or analysed during the current study.
