# Correction to: Pre-Transplant Immune Dysregulation Predicts for Poor Outcome Following Allogeneic Haematopoietic Stem Cell Transplantation in Adolescents and Adults with Inborn Errors of Immunity (IEI)

**DOI:** 10.1007/s10875-025-01893-z

**Published:** 2025-05-16

**Authors:** Thomas A. Fox, Valerie Massey, Charley Lever, Rachel Pearce, Arian Laurence, Sarah Grace, Filippo Oliviero, Sarita Workman, Andrew Symes, David M. Lowe, Valeria Fiaccadori, Rachael Hough, Susan Tadros, Siobhan O. Burns, Markus G. Seidel, Ben Carpenter, Emma C. Morris

**Affiliations:** 1 UCL Institute of Immunity and Transplantation, UCL, London, UK; 2 Department of Haematology, University College London Hospitals NHS Foundation Trust, London, UK; 3 Department of Immunology, Royal Free London Hospitals NHS Foundation Trust, London, UK; 4 Kings College London, London, UK; 5 Division of Pediatric Hematology-Oncology, Department of Pediatrics and Adolescent Medicine, Styrian Children’s Cancer Research Unit for Cancer and Inborn Errors of the Blood and Immunity in Children, Medical University of Graz, Graz, Austria

**Correction to: Journal of Clinical Immunology (2025) 45:64**

10.1007/s10875-024-01854-y

In the published version, there was an erratum in the abstract:

- In the first sentence, the word “disease” was used twice:

Incorrect: “Allogeneic haematopoietic stem cell transplantation (alloHSCT) is safe and effective for adolescents and adults with inborn errors of immunity (IEI) with severe disease manifestations of their disease.”

Corrected: “Allogeneic haematopoietic stem cell transplantation (alloHSCT) is safe and effective for adolescents and adults with inborn errors of immunity (IEI) with severe manifestations of their disease.”

- In the last sentence of the abstract, there was a typographical error in the word “regarding.”

The original version has been corrected.

