# Supplementary material for: Pre-Transplant Immune Dysregulation Predicts for Poor Outcome Following Allogeneic Haematopoietic Stem Cell Transplantation in Adolescents and Adults with Inborn Errors of Immunity (IEI)
Source: J Clin Immunol. 2025 Jan 6;45(1):64. doi: 10.1007/s10875-024-01854-y (PMC11703937; doi:10.1007/s10875-024-01854-y)
Supplement: Supplementary file 1 — Supplementary Material 1 [file 10875_2024_1854_MOESM1_ESM.docx]

**Supplementary table 1 – Components of IDDA v2.1 – adopted from Seidel *et al*, 2022^1^**

| **Parameters**  All graded as:  0 = absent, inactive  1 = mild, transient, not requiring treatment  2 = moderate, intermittent therapy needed  3 = severe, continuous therapy needed  4 = life-threatening, refractory, irreversible | |
| --- | --- |
| **1** | Autoimmune (AI) cytopenia |
| **2** | Haemophagocytosis \| HLH (according to clinical AND lab criteria of the HS) |
| **3** | Enteropathy \| IBD |
| **4** | Lymphoproliferation \| splenomegaly \| hepatomegaly |
| **5** | Parenchymal lung disease \| LIP \| GLILD |
| **6** | Skin or eye manifestations \| eczema, uveitis, alopecia, vitiligo, other |
| **7** | Granulomatous disease in any organ (except GLILD) |
| **8** | Endocrinopathy \| IDDM, thyreoiditis, other |
| **9** | Arthritis \| other musculoskeletal |
| **10** | AI-hepatitis \| cholangitis \| pancreatitis |
| **11** | Glomerulonephritis \| nephropathy, tubulopathy |
| **12** | Neurologic manifestations of immune dysregulation \| CNS autoimmunity, inflammation, vasculitis |
| **13** | Failure to thrive \| malresorption, wasting |
| **14** | Severe infections \| opportunistic (excl. chronic infestation, see below) |
| **Other factors and symptoms (will multiply or add to the IDDAscore)^2^** | |
| **15** | Karnovsky / Lansky Performance Scale (%) |
| **16** | Hospitalisation  (days out of 100 days; including day clinic stays, excl. intensive care unit) |
| **17** | Mechanical ventilation or other ICU measures  (days out of 100 days; except elective procedures) |
| **18** | Immunoglobulin substitution therapy \| hypogammaglobulinemia |
| **19** | Any relevant chronic or recurring infestation/infection (e.g., Norovirus, EBV) |
| **20** | Any other organ dysfunction / malady (e.g., cardiomyopathy, kidney failure) |
| **21** | Nutrition / dietary status and habits |
| **22** | Malignancy (separately noted, not added to numeric score) |
| Formula for IDDA2.1 score total (Excel® format)   = (SUM(line1:line14) + IF(line16 < 40;line16*0.1;4) + IF(line17 < 10;line17*0.8;8) + SUM(line18:line21))*IF(line15 > 29;150/line15;6) | |
| 1, grading for lines 1–14: 0, absent; 1, mild, transient, not requiring treatment; 2, moderate, intermittent therapy needed; 3, severe, continuous therapy needed; 4, life-threatening, refractory, irreversible  2, lines 15–17 are percentages, lines 18–21 are scored as follows: line 18 (0, no; 2, sporadic; 3[iv], regularly IVIG; 3[sc], regularly SCIG); line 19 (0, no; 1, asymptomatic infestation; 2, oligosymptomatic recurring infection; 3, recurring symptomatic infection requiring on/off treatment; 4, chronic infection requiring permanent treatment or refractory infection, only score worst if more than one microbial agents are relevant); line 20 (e.g., hepatopathy, cardiomyopathy, kidney failure; please quantify if possible: 0, no organopathy; 1, mild transient dysfunction; 2, chronic mild dysfunction; 3, moderate-severe dysfunction; 4, clinically compromising dysfunction requiring treatment or replacement therapy, only score worst if more than one organ is involved); line 21 (0, normal; 1, modified disease-adjusted; 2, part-formula medically advised; 3, tube-feeding and/or full-formula or partial parenteral nutrition (irregularly); 4, total parenteral nutrition) | |

**Supplementary Table 1 adapted from:**

1. Seidel MG, Tesch VK, Yang LN, et al. The Immune Deficiency and Dysregulation Activity (IDDA2.1 'Kaleidoscope') Score and Other Clinical Measures in Inborn Errors of Immunity (Nov, 10.1007/s10875-021-01177-2, 2021). *Journal of Clinical Immunology*. 2022;42(3):499-499.

**Supplementary table 2 – HCT-CI and IDDA scores for individual patients**

|  | **Diagnosis** | **Age at transplant** | **HCT-CI score** | **IDDA v2.1 score pre-transplant** |
| --- | --- | --- | --- | --- |
| **1** | Autoimmune LPD | 32 | 2 | Not available |
| **2** | XLPD, | 18 | 3 | Not available |
| **3** | CVID | 30 | 4 | 35 |
| **4** | CVID | 31 | 3 | Not available |
| **5** | X-CGD | 23 | 1 | 12 |
| **6** | Autoimmune LPD | 34 | 0 | 13.5 |
| **7** | X-CGD | 27 | 4 | Not available |
| **8** | Cgamma SCID | 26 | 3 | Not available |
| **9** | X-CGD | 18 | 1 | 20 |
| **10** | X-CGD | 14 | 1 | 12 |
| **11** | NK and IgG subclass deficiency | 27 | 1 | 4.5 |
| **12** | X-CGD | 14 | 1 | 32 |
| **13** | X-CGD | 19 | 1 | 29 |
| **14** | DCML/MonoMac Deficiency Gata2 splice site mutation | 27 | 0 | 12.75 |
| **15** | AR IL12Rbeta defic | 29 | 1 | 6.6 |
| **16** | X-CGD | 19 | 1 | 20 |
| **17** | Undefined SCID | 22 | 0 | 12.2 |
| **18** | Gata 2 Defic | 22 | 2 | Not available |
| **19** | AR CGD | 28 | 1 | 10.9 |
| **20** | AR CGD, | 19 | 1 | 23 |
| **21** | X-CGD | 13 | 3 | 28 |
| **22** | AR CGD | 18 | 0 | 10 |
| **23** | X-CGD | 17 | 2 | 9 |
| **24** | CVID, APDS2 | 50 | 2 | 18.75 |
| **25** | AR CGD | 27 | 2 | Not available |
| **26** | Rag2 Heterozygous; Red cell aplasia | 20 | 0 | 21 |
| **27** | X-CGD | 17 | 4 | 25 |
| **28** | XIAP | 20 | 1 | 13 |
| **29** | CD27 deficiency | 18 | 1 | 7 |
| **30** | Gata2 | 20 | 0 | 9 |
| **31** | Gata2 MDS | 23 | 0 | 12 |
| **32** | XIAP | 25 | 2 | 22 |
| **33** | FasL, Autoimmune LPD, HL | 20 | 0 | 10 |
| **34** | X-CGD, | 13 | 2 | 13.5 |
| **35** | AR CGD | 14 | 0 | 18 |
| **36** | Gata2 MDS | 24 | 0 | 19.5 |
| **37** | Hyper IgE (STAT3 LOF) | 19 | 1 | 22.5 |
| **38** | Genetically undefined CID | 18 | 0 | 9 |
| **39** | Genetcially undefined CID | 17 | 4 | 30 |
| **40** | CD40L Deficiency | 31 | 1 | 17 |
| **41** | Gata2 | 24 | 1 | 12 |
| **42** | APDS2/aPI3K syndrome | 16 | 2 | 20 |
| **43** | Genetically undefined CID | 28 | 0 | 36 |
| **44** | CARD 9 Deficiency | 51 | 1 | 35 |
| **45** | Gata2/MDS | 13 | 2 | 5.6 |
| **46** | HLH | 29 | 1 | 25 |
| **47** | DOCK8 Deficiency | 34 | 0 | 8 |
| **48** | X-CGD | 25 | 1 | 23 |
| **49** | LoCID | 56 | 5 | 23 |
| **50** | Hypomorphic Rag | 40 | 3 | 17 |
| **51** | CNS HLH | 16 | 5 | 19.5 |
| **52** | DOCK 8 Defic | 18 | 3 | 10.8 |
| **53** | APDS2/aPI3K syndrome | 17 | 6 | 20 |
| **54** | X-CGD | 35 | 3 | 10 |
| **55** | X-CGD | 28 | 4 | 17 |
| **56** | Genetically undefined CID | 17 | 0 | 6 |
| **57** | Genetically undefined CID | 26 | 5 | 42.5 |
| **58** | X-SCID (prior GT Dec 2005) | 15 | 2 | 46.75 |
| **59** | DOCK 8 Deficiency | 15 | 1 | 6.75 |
| **60** | CD40L Deficiency | 15 | 1 | 10.5 |
| **61** | CNS HLH; compound Het in PRF1 | 18 | 0 | 10.5 |
| **62** | X-CGD | 17 | 1 | 8.3 |
| **63** | Perf-/- HLH | 21 | 1 | 40 |
| **64** | GATA2 Deficiency | 59 | 1 | 13.5 |
| **65** | Job Syndrome/Hyper IgE; STAT3 mutation | 19 | 0 | 16.67 |
| **66** | X-SCID post GT (unconditioned gamma RV in infancy, Dec 2001) | 20 | 5 | 21.6 |
| **67** | X-CGD | 16 | 1 | 13.3 |
| **68** | WAS | 39 | 0 | 25 |
| **69** | WAS | 43 | 0 | 13.5 |
| **70** | CD40L Deficiency | 34 | 4 | 7.5 |
| **71** | Clericuzio Syndrome | 24 | 2 | 16.875 |
| **72** | CD40L Deficiency | 17 | 2 | 12 |
| **73** | DOCK8 Deficiency | 15 | 0 | 12 |
| **74** | XLA | 34 | 1 | 10.8 |
| **75** | X-CGD | 37 | 3 | 15 |
| **76** | AR-CGD | 14 | 1 | 15.5 |
| **77** | AR-CGD | 39 | 3 | 13.5 |
| **78** | DADA2 | 23 | 4 | 27.5 |
| **79** | STAT1 gain-of-function | 21 | 1 | 30.83 |
| **80** | X-CGD | 15 | 2 | 30 |
| **81** | CTLA4 Insufficiency | 56 | 6 | 50 |
| **82** | LAD syndrome type I partial | 14 | 2 | 16.6 |
